# Local Coverage Optimization Strategy Based on Voronoi for Directional Sensor Networks †

**DOI:** 10.3390/s16122183

**Published:** 2016-12-18

**Authors:** Guanglin Zhang, Shan You, Jiajie Ren, Demin Li, Lin Wang

**Affiliations:** 1 College of Information Science and Technology, Donghua University, Shanghai 201620, China; 2141077@mail.dhu.edu.cn (S.Y.); renjiajie@mail.dhu.edu.cn (J.R.); deminli@dhu.edu.cn (D.L.); 2 Engineering Research Center of Digitized Textile & Fashion Technology, Ministry of Education, Shanghai 201620, China; 3 Department of Automation, Shanghai Jiao Tong University, Shanghai 200240, China; wanglin@sjtu.edu.cn

**Keywords:** area coverage, Voronoi diagram, wireless sensor networks (WSNs), directional sensor networks (DSNs), angle of view (AoV)

## Abstract

In this paper, we study the area coverage of directional sensor networks (DSNs) with random node distribution. The coverage of DSNs depends on the sensor’s locations, the sensing radiuses, and the working directions, as well as the angle of view (AoV), which is challenging to analyze. We transform the network area coverage problem into cell coverage problems by exploiting the Voronoi diagram, which only needs to optimize local coverage for each cell in a decentralized way. To address the cell coverage problem, we propose three local coverage optimization algorithms to improve the cell coverage, namely Move Inside Cell Algorithm (MIC), Rotate Working Direction Algorithm (RWD) and Rotation based on boundary (RB), respectively. Extensive simulations are performed to prove the effectiveness of our proposed algorithms in terms of the coverage ratio.

## 1. Introduction

With the rapid development of information technologies, wireless sensor networks (WSNs) are now playing an increasingly important role in environment monitoring, disaster rescuing target tracking, and industrial process control, etc. Generally, the sensor nodes are deployed in a target area to collect environment information for further processing. Such a complex network calls for novel approaches for system design to make it operate efficiently.

The area coverage problem of WSNs is important in both theory and application. As a main measurement of the network quality of service (QoS), the coverage problem attracts a lot of attention and is a hot research topic [1,2]. Previous works focused on the omnidirectional sensor networks, where the sensor’s working coverage area is a circle. However, with the increasing demand and more diverse applications, WSNs have some disadvantages in terms of information access, e.g., in the applications of image/video sensing.

Due to its flexibility with rotation and lower energy consumption, directional sensor networks (DSNs) have been widely investigated in the literature. For a DSN, the directional sensor is limited to a certain working direction and the angle of view (AoV), such as camera sensors, infrared sensors, and so on. Different from omnidirectional WSNs, in which the sensing coverage mainly depends on sensing radius ($R_{s}$) and sensor location, in DSNs, sensing coverage is affected not only by the location and sensing radius ($R_{s}$) but also by the working direction and AoV. Furthermore, under the condition of random deployment, directional sensors can improve coverage dynamically by adjusting their working directions. Moreover, compared with the WSNs, the nodes in DSNs have the ability of directional sensing, which results in a lower energy consumption and mutual interference. Recently, the enormous application potential of DSNs attracts lots of attention from researchers [3,4,5,6]. In [3,4], the authors investigate how to utilize virtual force between adjacent sensors to improve the coverage. In [5,6], Voronoi tessellation is adopted to tackle the coverage problem. In our previous work [7], we utilize the combination of node’s mobility and motility to increase coverage.

In this paper, we adopt a Voronoi diagram to investigate the coverage problem of DSNs with random node deployment. The main characteristics of such an approach are: (i) by the construction of a Voronoi diagram for the random deployed sensors, each Voronoi cell contains one sensor; (ii) every sensor stores all of the vertices’ information of the Voronoi cell in which it is located, which is used for decision-making on node moving and rotation; and (iii) every sensor node’s actions (i.e., moving or rotation) should be within its related Voronoi cell.

The main contributions of this paper are as follows:

1. We transform the network area coverage problem into cell coverage problems by exploiting the Voronoi diagram, which only needs to optimize local coverage for each cell. The proposed approach solves the problem in a decentralized way.
2. By adopting a node’s moving or rotation actions, we propose three local coverage optimization algorithms to improve the cell coverage, i.e., Move Inside Cell Algorithm (MIC), Rotate Working Direction Algorithm (RWD), Rotation based on boundary (RB), respectively.
3. We use extensive simulations to prove the effectiveness of our proposed algorithms in terms of the coverage ratio. Specifically, compared to the benchmark algorithm Distributed Voronoi-Based SelRedeployment algorithm (DVSA) proposed in [8], our algorithm MIC shows a shorter moving distance and lower energy consumption.

The rest of this paper is organized as follows: we present the literature review in Section 2. In Section 3, we present the system model and problem statement. In Section 4, we show the theoretical analysis and propose the related algorithm. In Section 5, we give the performance evaluation results to demonstrate the effectiveness of our proposed algorithms. Conclusions and future works are shown in the final section.

## 2. Related Work

Many studies have been done on area coverage in both WSNs and DSNs [9,10]. For example, in [11,12], the authors studied the coverage of hybrid WSNs. They employed a Voronoi diagram to address the coverage problem. Specifically, they use static sensors to estimate coverage holes, work out assisted sensor’s positions in every cell, and order mobile sensors to heal coverage holes. Liang et al. [13] presented two distributed self-deployment schemes of mobile sensors, namely circumcenter-based and incenter-based. The first scheme constructs a Voronoi diagram by the circumcenter of the sensor’s sensing sector. If a sensor does not get full coverage, then a sensor moves until the circumcenter coincides with the centroid of its Voronoi cell. The second scheme uses a similar pattern, and the main difference is that it employs an incenter of the sensor’s sensing sector to construct a Voronoi diagram.

In [3,4], the authors presented coverage enhanced algorithms based on virtual force between sensors. Tao et al. [4] presented a potential field based coverage-enhancing algorithm (PFCEA). PFCEA mainly takes a centroid of the sensing sector area as a stressed point, and the sensor adjusts its working direction by a resultant force from neighbor sensors. Liang et al. [3] also employed virtual force, and the difference is that they added a Voronoi diagram based on [4] and proposed a scheme that consists of four different forces caused by neighboring sensors and uncovered regions in the field, namely, the Centroid Push Auxiliary point Force (CPAF), the Centroid Push Centroid Force (CPCF), the Voronoi point Pull Centroid Force (VPCF), and the Neighbor Repulsive Force (NRF). The main idea of their work is to adjust working direction based on resultant force of sensor. Lin et al. [14] proposed two enhanced deployment algorithms, EDA-I and EDA-II, respectively. EDA-I adopts the concept of virtual force to instruct the sensor to move or rotate, and EDA-II constructs the Voronoi diagram by the centroid of the sensor’s fan. If the sensor does not cover all Voronoi vertices surrounding its cell, then the sensor moves until the centroid of the fan coincides with the cell’s centroid. Actually, in EDA-II, it is hard to guarantee that all vertices surrounding cells are covered by the sensor itself, which means that all sensors need to move in the scheme of EDA-II. In addition, they proposed scheme Virtual Boundary Torques (VBT) to solve the problem of sensors whose location is outside the boundary. However, they did not solve the problem of whose location is within the boundary but effective working area towards outside the border.

Wen et al. [8,15] adopted Voronoi and Delaunay triangulation theory to design a distributed self-redeployment coverage enhancement algorithm. In [8], they proposed DVSA based on the idea that calculates corresponding field angles and side lengths of each vertex side in each polygon, and then compares AoV and $R_{s}$ with field angles and side lengths. If there is no difference between the results, then a sensor moves to that vertex and takes the longer side as one boundary of the sensing sector. In [15], they presented several coverage increment algorithms, vertex-based adjustment with Voronoi diagram (V-VD), edge-based adjustment with Voronoi diagram (E-VD), edge-based adjustment with Delaunay triangulation (E-DT), and angle-based adjustment with Delaunay triangulation (A-DT). In E-VD, the sensor chooses the midpoint of the farthest edge of its own cell as a working direction. In E-DT, the authors utilized sensors to construct Delaunay triangulation, the sensor chooses the midpoint of the farthest edge of its own triangulation as a working direction, and, in A-DT, the sensor chooses the vertex with the maximum angle as the working direction.

In this paper, we study the area coverage of DSNs in the case of random deployment. The sensor makes a decision to adjust working direction and update location based on the vertex information of its Voronoi cell. The goal is to get local maximal coverage in the cell in which the sensor is located.

## 3. System Model and Problem Statement

### 3.1. DSN Sensing Model

**Figure 1:**
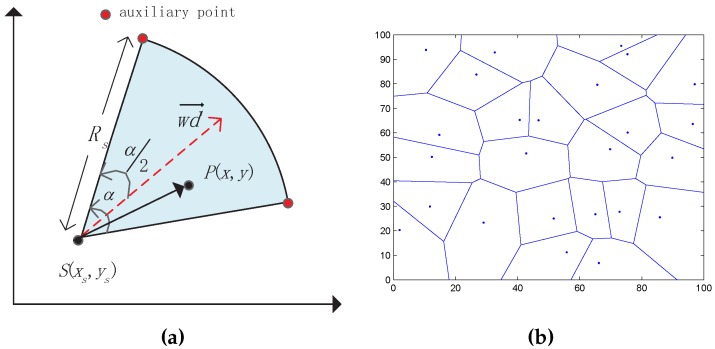
Sensing model of directional sensor networks (DSN) and Voronoi diagram (**a**) Sensing model; (**b**) Voronoi diagram.

As shown in Figure 1a, a directional sensor is characterized by the directional working direction *ω* (-π≤ω≤π) and the AoV *α*. A unit vector $\vec{wd}$ is defined as the working direction which splits the angle *α*. The effective coverage field of sensor is the sector area $\alpha R_{s}^{2}/2$. We introduce two auxiliary points $a_{l}$, and $a_{r}$ to help sensors make decisions. We said that a point p(x,y) is covered by a sensor $s(x_{s},y_{s})$, if the following two conditions are satisfied:

1. Euclidean distance between *p* and *s* must be smaller than sensing radius $R_{s}$, i.e., $d\left(s,p\right)\leq R_{s}$:
  (1) $$d\left(s,p\right)=\sqrt{{\left(x-x_{s}\right)}^{2}+{\left(y-y_{s}\right)}^{2}}\leq R_{s}.$$
2. The absolute included angle between $\vec{wd}$ and $\vec{sp}$ must be smaller then α/2:
  (2) $$\phi =\arccos\frac{\vec{wd}\cdot \vec{sp}}{∥sp∥}\leq \frac{\alpha }{2}.$$

### 3.2. Voronoi Diagram and Some Assumptions

A Voronoi diagram is an important data structure in computational geometry that is widely used in many fields [16,17]. The main properties we need to use in this paper are that: (1) every sensor node is located at the Voronoi cell that is constructed in the initiation and any point *p* within the $s_{i}^{\prime }s$ cell has the shortest distance between sensor $s_{i}$ and point *p* compared to other sensors, i.e., $d\left(s_{i},p\right)\leq d\left(s_{j},p\right)$; (2) a point in a shared Voronoi edge of two sensors has the same distance from two sensors; and (3) each sensor knows the vertex coordinate of its corresponding cell according to the related distributed/localized Voronoi algorithm. Given *N* sensors $s_{1}$, $s_{2}$, ..., $s_{N}$, we divide it into *N* cells based on the Voronoi principle. As shown in Figure 1b, we choose 25 random nodes as an example to generate a Voronoi diagram.

We make the following assumptions in this paper to simplify the problem analysis:

- All of the directional sensors are homogeneous, that is to say, every sensor has the same sensing radius $R_{s}$, viewing angle *α*, rotation ability and mobility.
- We can obtain an accurate coordinate of every sensor node by using Global Position System (GPS) or other alternative localization algorithms such as DV-hop, Amorphous, etc.
- All of the sensor nodes have strong transmission ability to ensure the network connectivity and Voronoi diagram constructed successfully.

### 3.3. Problem Statement

We aim to maximize the effective coverage for the target area by utilizing a sensor node’s rotation and moving action in the DSNs. The main difficulties that we face is that there exists coverage holes and overlap between a sensor node’s sensing area. That is to say, we try to control the sensor node’s actions in terms of rotation and movement to reduce the overlap and enlarge the effective coverage. We assume that the sensor’s mobility and working direction adjustment should satisfy the following conditions.

**Figure 2:**
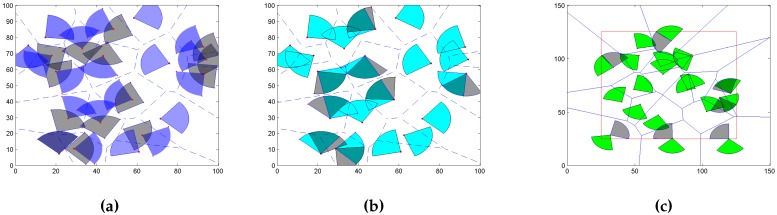
Example of mobility and rotation. (**a**) Move Inside Cell Algorithm (MIC); (**b**) Rotate Working Direction Algorithm (RWD); (**c**) Rotation based on boundary (RB).

Here, we give a simple exhibition of coverage algorithms that we have designed as shown in Figure 2. Figure 2a illustrates the mobility deployment, Figure 2b illustrates the rotation deployment, and Figure 2c illustrates the boundary repair deployment, and the grey area is the status after algorithms. The key part of our designed algorithms lies in taking advantage of the Voronoi information to adjust working directions or update locations. The main goal of a node’s movement or rotation is to let the sensor get full coverage (sector working area is completely in the cell) in its own cell and reduce overlap with other sensors:

- Restrict the sensor node’s moving trace in its corresponding cell.
- Get maximal coverage in each cell and minimum overlap with another cell’s sensor fan.
- Maximize the cell coverage under the constraints of minimized moving distance.

## 4. Theoretical Analysis and Algorithms

In this section, we propose the theoretical analysis and the corresponding algorithms. We take the advantage of the Voronoi tessellation to divide the network area into Voronoi cells based on the locations of deployed sensors. However, the initial coverage is not optimal in the case of random node distribution, Here, we consider whether the sensor in each cell gets the maximum coverage. If not, the sensor will update location or adjust $\vec{wd}$.

### 4.1. To Determine Whether a Sensor’s Sector Area is Wrapped in the Cell (Gets Full Coverage)

**Figure 3:**
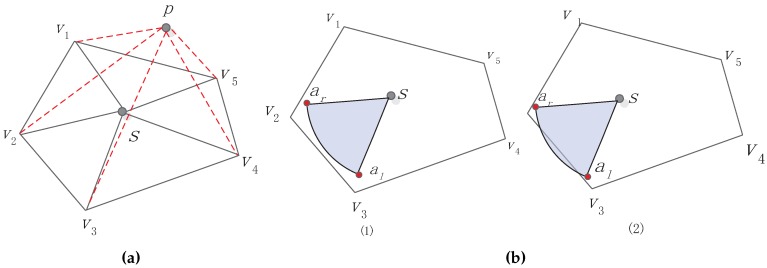
Proof point in convex polygon and sector wrapped in convex polygon. (**a**) Point in polygon; (**b**) sector wrapped in convex polygon.

If the sensor *s* and its auxiliary points $a_{l}$ and $a_{r}$ are all inside the polygon as shown in Figure 3(b1) and Figure 3(b2),then we say that the sensor *s* gets full coverage. In addition, the sensor does not need to move or rotate.

To prove that sensor *s*’s working area is wrapped in the cell, we can prove that points *s*, $a_{l}$ and $a_{r}$ are all in the cell, which is a convex polygon. To prove whether one point (assuming *p*) is in the convex polygon, we employ triangle segmentation to compute the convex polygon area $S_{v_{s}}$ and the area $S_{pv_{s}}$ constructed by *p* and *V*, as Figure 3a demonstrates. Here, $v_{s}$ denotes a vertex set of sensors:

(3) $$S_{v_{s}}=\sum_{v_{i=1}}^{V_{s}-1}S_{\Delta sv_{i}v_{i+1}}\;\;\;\;\;S_{pv_{s}}=\sum_{v_{i=1}}^{V_{s}-1}S_{\Delta pv_{i}v_{i+1}},$$

where $S_{v_{s}}$ represents the area of each cell (convex polygon) and $S_{pv_{s}}$ represents the area of polygon constructed by the certain point *p* and cell vertex. We use Equation (3) to judge whether one point is in the cell. If $S_{v_{s}}=S_{pv_{s}}$, then we can know that the point *p* is inside the convex polygon. Based on this idea, if two auxiliary points $a_{l}$ and $a_{r}$ of sensor *s* are both in the polygon, we can infer that the sensor gets relatively full coverage, although not completely, as shown in Figure 3(b2). If $S_{v_{s}}\neq S_{pv_{s}}$, which means that the sensor does not get full coverage in its own cell, then the sensor needs to move or adjust $\vec{wd}$.

### 4.2. Move and Rotate inside the Cell Based on the Vertex

**Figure 4:**
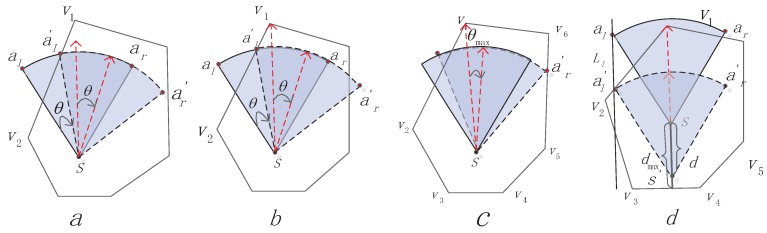
Move and rotate inside the cell. (**a**) rotate; (**b**) rotate $a_{r}$ leads $a_{r}^{\prime }$; (**c**) compute the $\theta_{max}$; (**d**) compute $d_{max}$ and *d*.

If one auxiliary point ($a_{l}$ or $a_{r}$ ) of the sensor node is not inside the cell, the location or working direction will adjust. We choose the farthest vertex (assume $v_{1}$) of the polygon as the $s^{\prime }s$ initial working direction, which makes the sensor node get full coverage with a high chance. We find that this decision will lead to minimum moving distance or the rotation angle getting full coverage. Let us look at Figure 4a. First, the sensor selects the farthest vertex $v_{1}$ as the working direction $\vec{sv_{1}}$, and there are two situations here:

#### 4.2.1. Case 1: Rotate Working Direction Algorithm (RWD)

$R_{s}$ ≤ $d(s,v_{1})$ and only one auxiliary point ($a_{r}$ or $a_{l}$) is inside its own cell.

For this situation, we can rotate the working direction by *θ* to get the aim of letting the $a_{l}^{\prime }s$ position update to $a_{l}^{\prime }$. Rotating *θ* may lead $a_{r}^{\prime }$ to be out of the polygon, as Figure 4b shows. Therefore, we should primarily figure out $\theta_{max}$ (see Figure 4c), which is the maximal rotation angle. If $\theta \leq \theta_{max}$, the sensor rotates the working direction by *θ*. If not, it rotates by $\theta_{max}$.

We state how to compute *θ* and $\theta_{max}$ in the following: according to $s(x_{s},y_{s})$ and the corresponding angle *ω* of $\vec{wd}$, we can get the included angle *β* of $L_{sa_{l}^{\prime }}$ relative to the coordinate axis and coordinate of $a_{l}^{\prime }$:

(4) $$\psi =\arctan(\frac{y_{v_{1}}-y_{s}}{x_{v_{1}}-x_{s}})\;\;\;\;,$$

if *ψ* ≤ 0,

(5) $$\begin{array}{c} \;\;\;\;\;\;\;\;\;\;\;\;\;\left\{\begin{array}{ccc} \omega =\psi & & x_{v_{1}}\geq x_{s},y_{v_{1}}\leq y_{s}\\ \omega =\psi +\pi & & x_{v_{1}}<x_{s},y_{v_{1}}>y_{s} \end{array}\right. \end{array},$$

if *ψ* ≥ 0,

(6) $$\;\;\;\;\;\;\;\;\;\;\;\;\;\left\{\begin{array}{ccc} \omega =\psi & & x_{v_{1}}\geq x_{s},y_{v_{1}}\geq y_{s}\\ \omega =\psi +\pi & & x_{v_{1}}<x_{s},y_{v_{1}}<y_{s} \end{array}\right.,$$

if sensor rotates right,

(7) $$\left\{\begin{array}{ccc} \beta =\omega +\frac{\alpha }{2}-\theta & & \omega \geq 0\\ \beta =\omega -\frac{\alpha }{2}+\theta & & \omega <0 \end{array}\right.,$$

if sensor rotates left,

(8) $$\left\{\begin{array}{ccc} \beta =\omega -\frac{\alpha }{2}+\theta & & \omega \geq 0\\ \beta =\omega +\frac{\alpha }{2}-\theta & & \omega <0 \end{array}\right..$$

We can get the *θ* by solving Equation (9):

(9) $$\;\;\;\;\;\;\;\;\;\;\;\;\;\;\;\;\left\{\begin{array}{ccc} x_{a_{1}^{\prime }} & = & x_{s}+R_{s}\cos\left(\beta \right)\\ y_{a_{1}^{\prime }} & = & y_{s}+R_{s}\sin\left(\beta \right)\\ L_{v_{1}v_{2}} & = & \frac{y_{v_{1}}-y_{v_{2}}}{x_{v_{1}}-x_{v_{2}}}\left(x-x_{v_{1}}\right)+y_{y_{1}} \end{array}\right..$$

In a similar way, as shown in Figure 4c, we can solve $\theta_{max}$ according to the above method, and the only difference is that we combine $a_{r}^{\prime }$ with line $L_{v_{5}v_{6}}$.

#### 4.2.2. Case 2: Move Inside Cell Algorithm (MIC)

Two auxiliary points of the sensor *s* are both outside the polygon, Figure 4d.

For this situation, the sensor cannot achieve the goal of getting full coverage if only by rotation, so the sensor should move, and the moving direction and moving distance are primarily under consideration. We choose the reverse direction of $\vec{sv_{1}}$ as the moving direction and take $a_{l}$, which is far from polygon boundary, as the reference point. If $a_{1}$ is moved into the polygon, then $a_{r}$ is also in the polygon. Moving distance $d=d\left(a_{l},a_{l}^{\prime }\right)=d\left(s,s^{\prime }\right)$, as shown in Figure 4d. If $d\leq d_{max}$, the sensor moves by *d*. If not, it moves by $d_{max}$. If we can solve the coordinate of $a_{l}^{\prime }$, then the moving distance $d=d(a_{l},a_{l}^{\prime })$. Each node stores the vertex information of its own cell according to the related Voronoi generation method. $a_{l}^{\prime }$ is the intersection of line $L_{1}$ and $L_{v_{1}V_{2}}$. Then, if we can find out the equations of line $L_{1}$ and $L_{v_{1}V_{2}}$, the problem will be solved. Constructed calculation of *d* and $d_{max}$ is as below:

(10) $$\;\;\;\;\;\;\;\;\;\;\;\;\;\left\{\begin{array}{ccc} x_{a_{1}} & = & x_{s}+R_{s}cos\left(\omega +\frac{\alpha }{2}\right)\\ y_{a_{1}} & = & y_{s}+R_{s}sin\left(\omega +\frac{\alpha }{2}\right)\\ L_{1} & = & \frac{y_{v_{1}}-y_{v_{s}}}{x_{v_{1}}-x_{v_{s}}}\left(x-x_{a_{1}}\right)+y_{a_{1}}\\ L_{v_{1}v_{2}} & = & \frac{y_{v_{1}}-y_{v_{2}}}{x_{v_{1}}-x_{v_{2}}}\left(x-x_{v_{1}}\right)+y_{v_{1}} \end{array}\right..$$

We can get the coordinate of $a_{1}^{\prime }$ by combining $L_{1}$ with $L_{v_{1}v_{2}}$. Similarly, $d_{max}$ can be worked out by combining $L_{ss^{\prime }}$ with $L_{v_{3}v_{4}}$. The updated sensor location $\left(x_{s}^{\prime },y_{s}^{\prime }\right)$ is:

(11) $$x_{s}^{\prime }=\left\{\begin{array}{ccc} x_{s}-d_{max}cos\left(\omega \right) & & d_{max}\leq d\\ x_{s}-dcos\left(\omega \right)\;\;\;\;\; & & d_{max}>d \end{array}\right.,$$

(12) $$y_{s}^{\prime }=\left\{\begin{array}{ccc} y_{s}-d_{max}sin\left(\omega \right) & & d_{max}\leq d\\ y_{s}-dsin\left(\omega \right)\;\;\;\;\; & & d_{max}>d \end{array}\right..$$

**Figure 5:**
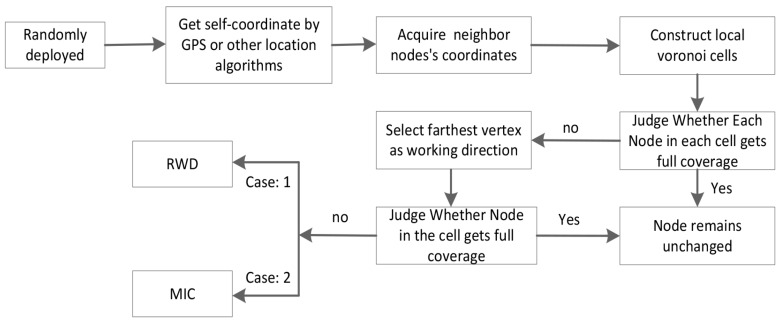
Procedures of the proposed decentralized local coverage optimization algorithm.

As shown in Figure 5, there are several major steps of the local coverage optimization algorithm, and the most worthwhile point is that not only can MIC and RWD run independently, but they can also run in combination with RB, and, just as later simulations reveal, the sensor employs the movement followed by rotation and finally calls the RB.

**Algorithm 1** Rotate Working Direction Algorithm (RWD)
1:$S_{s_{i}}$ **:** the area of the sensor.2:$S_{v_{s_{i}}}$ **:** denotes convex polygon area of the current sensor3:**Input:** location (X,Y) and $\vec{wd}$.4:**Output:** location and $\vec{wd}$.5:**Initialization:** Construct local Voronoi cell6:**while** i≤N **do**7:  **if** $S_{s_{i}}$ $\in S_{v_{s_{i}}}$ **then**8:      save $s_{i}\left(x_{s},y_{s}\right)$ and corresponding $\vec{wd}$9:  **else**10:      select $\vec{s_{i}v_{farhtest}}$ as $\vec{wd}$11:      **if** $S_{s_{i}}\in S_{v_{s_{i}}}$ **then**12:            save $s_{i}\left(x_{s}.,y_{s}\right)$ and corresponding $\vec{wd}$13:      **else** **if** $R_{s}\leq d\left(s_{i},v_{farthest}\right)$ && ($a_{l}$ $\notin S_{v_{s_{i}}}$) **then**14:            **if** $\theta \geq \theta_{max}$ **then**15:                    $\vec{wd}$ rotate to the right by $\theta_{max}$16:            **else**17:                    $\vec{wd}$ rotate to the right by *θ*18:            **end** **if**19:      **else** **if** $R_{s}\leq d\left(s_{i},v\right)$ && ($a_{r}$ $\notin S_{v_{s_{i}}}$) **then**20:            **if** $\theta \geq \theta_{max}$ **then**21:                    $\vec{wd}$ rotate to the left by $\theta_{max}$22:            **else**23:                    $\vec{wd}$ rotate to the left by *θ*24:            **end** **if**25:      **end** **if**26:  **end** **if**27:**end** **while**


**Algorithm 2** Move Inside Cell Algorithm (MIC)
1:**Input:** coordinates (X,Y) and $\vec{wd}$.2:**Output:** updated coordinates and $\vec{wd}$.3:**Initialization:** Construct local Voronoi cell4:**while** i≤N **do**5:  **if** $S_{s_{i}}$ $\in S_{v_{s_{i}}}$ **then**6:      save $s_{i}\left(x_{s},y_{s}\right)$ and corresponding $\vec{wd}$7:  **else**8:      select $\vec{s_{i}v_{farthest}}$ as $\vec{wd}$9:      **if** $S_{s_{i}}\in S_{v_{s_{i}}}$ **then**10:            save $s_{i}\left(x_{s}.,y_{s}\right)$ and corresponding $\vec{wd}$11:      **else** **if** ($a_{l},a_{r}$ $\notin S_{v_{s_{i}}}$) **then**12:            **if** $d\geq d_{max}$ **then**13:                    *s* moves $d_{max}$ to $s^{\prime }$14:            **else**15:                    *s* moves *d* to $s^{\prime }$16:            **end** **if**17:      **end** **if**18:  **end** **if**19:**end** **while**


### 4.3. Rotation Based on Boundary (RB)

From the above analysis, we know that the MIC and RWD can improve the area coverage. However, the performance may not be good enough for sensors that are deployed on the boundary. A sensor makes a decision to move or rotate based on whether auxiliary points are in its own polygon. In Figure 2c, we can see that boundary Voronoi cells are infinite, while, for the infinite cell, it is impossible for the sensor to decide whether auxiliary points are in the cell. Thus, the sensor cannot make a decision to move or rotate.

**Figure 6:**
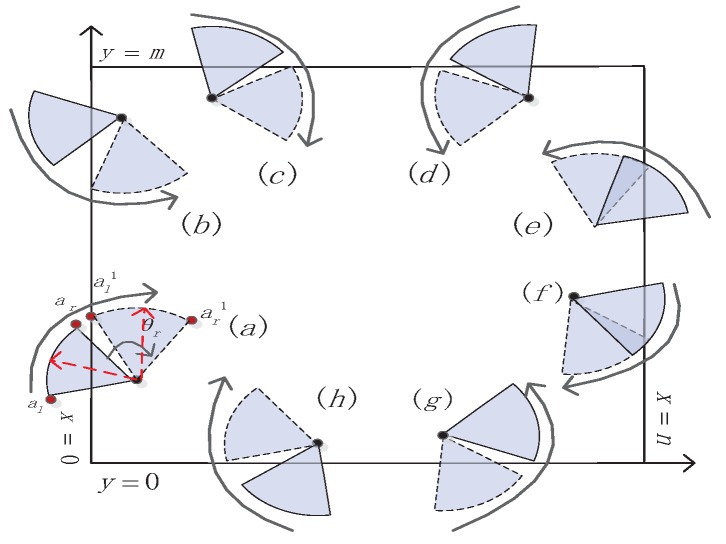
Rotation based on boundary. (**a**) boundary x=0, rotate right; (**b**) boundary x=0, rotate left; (**c**) boundary y=m, rotate right; (**d**) boundary y=m, rotate left; (**e**) boundary x=n, rotate left; (**f**) boundary x=n, rotate right; (**g**) boundary y=0, rotate left; (**h**) boundary y=0, rotate right.

For this case, we propose a scheme of rotation based on boundary (RB) to enhance boundary coverage. For a given target area, A=m×n and the sensor coordinate $s(x_{s},y_{s})$, $0\leq x_{s}\leq n$, $0\leq y_{s}\leq m$. The boundary sensor makes a decision to rotate according to whether the coordinates of the auxiliary points are inside the target area. In the process of rotation, we need to figure out the minimum rotation angle $\theta_{r}$, shown in Figure 6. To solve $\theta_{r}$, we take Figure 6a as an example. After rotation, $a_{l}$ updates to $a_{l}^{\prime }$, so $x_{a_{l}^{\prime }}=x_{s}+R_{s}\cos\left(\psi -\theta_{r}\right)$. We can get $\theta_{r}$ for $x_{a_{1}^{\prime }}=0$, and other cases can deduce from this. If the sensor rotates, $\theta_{r}$ can achieve the purpose. Then, the sensor rotating contrarily by a certain angle can also achieve the same purpose. From the perspective of energy consumption, the sensor needs to rotate a minimum angle that corresponds to several conditions as follows:

for boundary x=0,
  $$\;\;\;\;\;\;\;\;\;\;\left\{\begin{array}{cc} x_{a_{l}}\leq x_{a_{r}}\;\mathrm{and}\;x_{a_{l}}\leq 0 & \mathrm{rotate}\;\mathrm{right}\\ x_{a_{l}}<0\;\;\;\mathrm{and}\;x_{a_{r}}\geq 0 & \mathrm{rotate}\;\mathrm{right} \end{array}\right.,$$
  $$\;\;\;\;\;\;\;\left\{\begin{array}{cc} x_{a_{r}}\leq x_{a_{l}}\;\mathrm{and}\;x_{a_{r}}\geq 0 & \mathrm{rotate}\;\mathrm{left}\\ x_{a_{l}}\geq 0\;\;\;\mathrm{and}\;x_{a_{r}}<0 & \mathrm{rotate}\;\mathrm{left} \end{array}\right.,$$
for boundary x=n,
  $$\;\;\;\;\;\;\;\;\;\;\left\{\begin{array}{cc} x_{a_{l}}\leq x_{a_{r}}\;\;\mathrm{and}\;x_{a_{l}}\leq n & \mathrm{rotate}\;\mathrm{right}\\ x_{a_{l}}\geq n\;\;\;\;\mathrm{and}\;x_{a_{l}}<n & \mathrm{rotate}\;\mathrm{right} \end{array}\right.,$$
  $$\;\;\;\;\;\;\;\;\left\{\begin{array}{cc} x_{a_{r}}\geq x_{a_{l}}\;\;\mathrm{and}\;x_{a_{l}}\geq n & \mathrm{rotate}\;\mathrm{left}\\ x_{a_{l}}<n\;\;\;\;\mathrm{and}\;x_{a_{r}}\leq n & \mathrm{rotate}\;\mathrm{left} \end{array}\right.,$$
for boundary y=0,
  $$\;\;\;\;\;\;\;\;\;\left\{\begin{array}{cc} y_{a_{l}}\leq y_{a_{r}}\;\;\mathrm{and}\;y_{a_{r}}\leq 0 & \mathrm{rotate}\;\mathrm{right}\\ y_{a_{l}}\leq 0\;\;\;\;\mathrm{and}\;y_{a_{r}}>0 & \mathrm{rotate}\;\mathrm{right} \end{array}\right.,$$
  $$\;\;\;\;\;\;\;\;\;\left\{\begin{array}{cc} y_{a_{r}}\leq y_{a_{l}}\;\;\mathrm{and}\;y_{a_{l}}\leq 0 & \mathrm{rotate}\;\mathrm{left}\\ y_{a_{r}}\leq 0\;\;\;\;\mathrm{and}\;y_{a_{l}}>0 & \mathrm{rotate}\;\mathrm{left} \end{array}\right.,$$
for boundary y=m
  $$\;\;\;\;\;\;\;\;\;\;\left\{\begin{array}{cc} y_{a_{r}}\leq y_{a_{l}}\;\;\;\mathrm{and}\;y_{a_{r}}\geq m & \mathrm{rotate}\;\mathrm{right}\\ y_{a_{r}}<m\;\;\;\;\mathrm{and}\;y_{a_{l}}\geq m & \mathrm{rotate}\;\mathrm{right} \end{array}\right.,$$
  $$\;\;\;\;\;\;\;\left\{\begin{array}{cc} y_{a_{l}}\leq y_{a_{r}}\;\;\;\mathrm{and}\;y_{a_{l}}\geq m & \mathrm{rotate}\;\mathrm{left}\\ y_{a_{l}}<m\;\;\;\;\mathrm{and}\;y_{a_{r}}\geq m & \mathrm{rotate}\;\mathrm{left} \end{array}\right..$$

## 5. Performance Evaluation

In this section, we conduct extensive simulations to validate the efficiency of the algorithms that we proposed. We compare RWD, MIC and RB with random deployment, DVSA deployment in [8] and EDA-II deployment in [14] because DVSA and EDA-II both employ the movement and rotation characteristics. In addition, we analyze the impact of the sensing radius and AoV on network coverage ratio. For comparison of our proposed algorithm and the random deployment, we also compare the moving track of MIC with DVSA and EDA-II. Some essential simulation parameters are listed in Table 1.

### 5.1. Sensing Coverage

**Figure 7:**
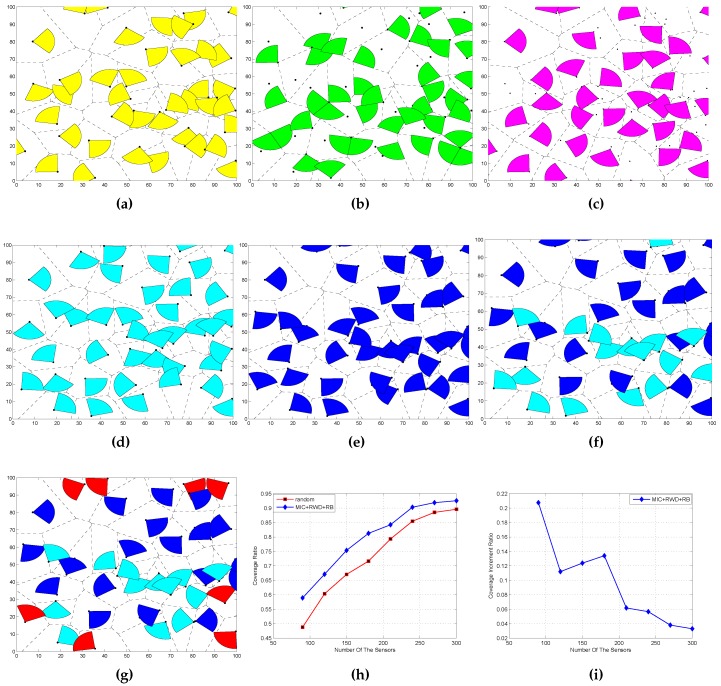
Coverage. (**a**) random; (**b**) DVSA; (**c**) EDA-II; (**d**) RWD; (**e**) MIC; (**f**) MIC and RWD; (**g**) MIC + RWD + RB; (**h**) Coverage ratio with $R_{s}=10$, AoV = $90^{\circ }$; (**i**) Coverage increment ratio with $R_{s}=10$, AoV = $90^{\circ }$.

In order to reflect the operation effect of algorithms that we have designed, we take the number of sensor N= 40, $R_{s}=10$ m and AoV is $90^{\circ }$. We compare our algorithms with random deployment, EDA-II in [14] and DVSA in [8]. Figure 7a is the random deployment and Figure 7b is the DVSA deployment, Figure 7c is EDA-II deployment, Figure 7d is the RWD deployment, Figure 7e is MIC deplyment and Figure 7f is the RWD + MIC. The last one, Figure 7g, is RWD + MIC + RB. By comparison, we can find that the combined algorithms gain an optimal coverage effect. RWD and MIC are based on the Voronoi cell, and, for the sensors not deployed at boundaries, they work well. RB is based on the boundary, and it can help the sensor deployed at the boundary rotate its working direction to the inner border, and this action increases node utilization.

Figure 7h shows the coverage ratio with respect to the number of sensor nodes *N* for fixed $R_{S}$ and AoV, while Figure 7i shows the coverage ratio increment with respect to *N* for fixed $R_{S}$ and AoV. Here, the coverage ratio increment is defined as $\frac{r_{\mathrm{algorithm}}-r_{\mathrm{random}}}{r_{\mathrm{random}}}$, where $r_{\mathrm{algorithm}}$ is the coverage ratio after execution of our proposed algorithm and $r_{\mathrm{random}}$ is the coverage ratio of the random deployment algorithm. We find that, in Figure 7i, the coverage increment ratio is decreasing with relatively large *N*. This is because the method of MIC and RWD are both based on a Voronoi cell, and the generated Voronoi cell is small when *N* is large, which results in a bigger working area of the sensor node than the Voronoi cell. Thus, it is hard for the sensor nodes to get full coverage in the cell by actions of movement or rotation. Because of this, the parameters of *N*, $R_{s}$, and AoV jointly affect the coverage ratio. In order to give a more clear illustration, in what follows, we investigate the coverage ratio with the three parameters, respectively.

### 5.2. Comparison from Moving Distance

**Figure 8:**
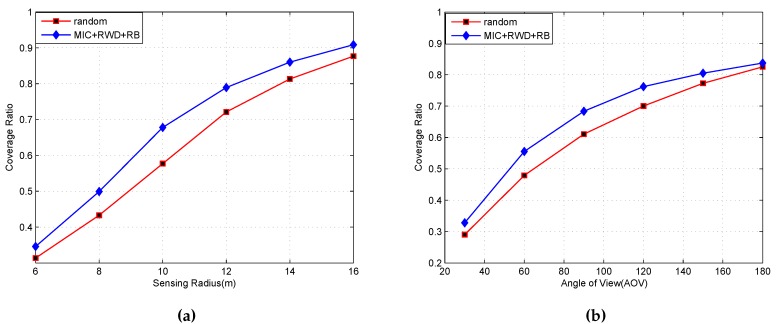
The influence of $R_{s}$ and AoV on coverage ratio. (**a**) MIC + RWD + RB compared with random(n = 120, AoV = $90^{\circ }$, $R_{s}$ = 6 m, 8 m,..., 16 m); (**b**) MIC + RWD + RB compared with random (n = 120, $R_{s}$ =10 m, AoV = $30^{\circ }$, $60^{\circ }$,..., $180^{\circ }$).

The moving distance is the main part of the node’s energy consumption. Here, we adopt the moving distance as the only measurement of the node’s energy consumption, for simplicity of analysis. Our algorithm can also extend to more general scenarios on energy consumption after slight changes. In DVSA, the sensor moves to the vertex with a side length and field angle similar to $R_{s}$ and AoV. In EDA-II, if the sensor does not cover all Voronoi vertices surrounding its own cell, then the sensor needs to move, while the MIC sensor makes a decision to move based on whether the auxiliary points are in the cell itself. Figure 8a,b,c represents the moving track of DVSA, EDA-II and MIC. The blue point is the initial position and the red point is the updated position. Clearly, we can see that the moving distance of MIC and EDA-II are shorter than DVSA. By comparing DVSA and EDA-II with MIC, we can find that the first two need all sensors to move despite moving distance being relatively shorter in EDA-II. However, not all sensors need to move in MIC, and network topology evolution is small.

### 5.3. Coverage Ratios with Different $R_{s}$ and AoV

In this section, we investigate the influence of different radii and angles of view on coverage. Figure 8 is the simulation result at different $R_{s}$ and AoV. In Figure 8a, we can see that when $R_{s}$ is smaller, coverage improvement is not very obvious. With $R_{s}$ increasing, the degree of improvement increases, and when $R_{s}$ is big enough, the improvement gradually disappears. The same process happens in Figure 8b. This is understandable because MIC and RWD make sensors move or rotate based on whether auxiliary points are in the cell. When $R_{s}$ and AoV increase to large enough sizes, all of the sensors may move or rotate, but this action blue does not, in fact, increase coverage. Thus, selecting suitable AoV and $R_{s}$ is important.

### 5.4. Coverage Ratio

**Figure 9:**
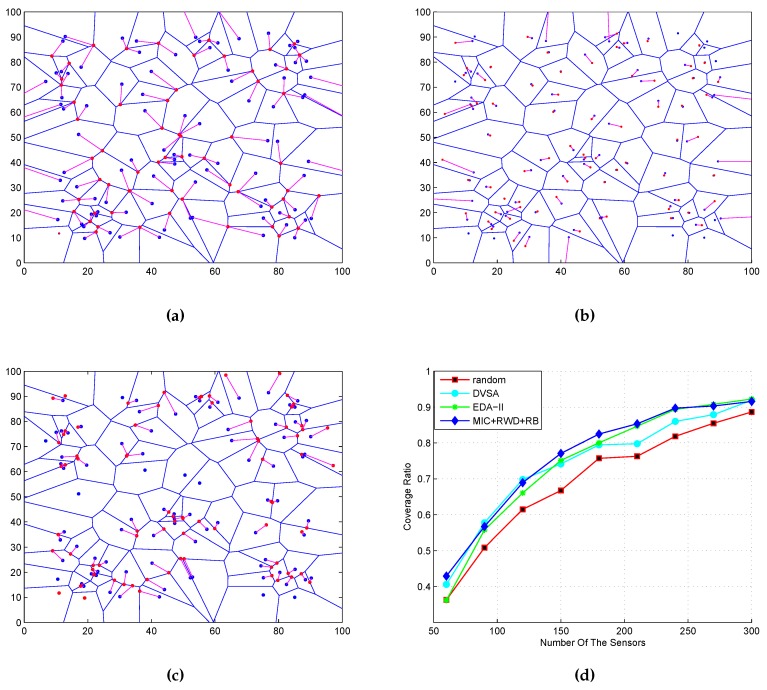
Moving track and coverage ratio. (**a**) DVSA; (**b**) EDA-II; (**c**) MIC; (**d**) coverage ratio.

Coverage ratio is a basic measure of DSNs. Figure 9d shows four kinds of coverage ratios that correspond to random deployment: DVSA, EDA-II, MIC coupled with RWD, and RB. To calculate the coverage ratio, we first regularly discrete the network area into *M* points. We sum all of the points that are covered by the sensors, which is denoted as *k*. Then, we can calculate the coverage ratio as k/M. Specifically, in the simulations, we first define a parameter *k*. If a point is covered by the sensor according to the criterion in Section 3.1, Equations (1) and (2), then k=k+1. Finally, we get the coverage ratio k/M. From the simulation results, we can see that MIC + RWD + RB get the better result. When the number of sensors *N* is less, the coverage ratio is not so remarkable, and, with *N* increasing, the coverage ratio is obvious. If *N* is big enough, then the advantage of the coverage increment ratio disappears, as shown in Figure 7i. However, this situation is foreseeable and understandable.

## 6. Conclusions

In this paper, we studied the area coverage problem of DSNs. We adopted the Voronoi diagram technique to transform the area network coverage problem into a cell coverage problem. We proposed three local coverage optimization algorithms to improve the cell coverage, i.e., MIC, RWD, and RB, respectively. We stated that algorithms have decentralized nature in the sense that sensors only need to construct their local Voronoi cells. We performed extensive simulations to prove the effectiveness of our proposed algorithms in terms of the coverage ratio. Compared to the benchmark algorithm DVSA that was proposed in the literature, we found that our proposed algorithm shows higher area coverage ratio and shorter moving distance. We also found that there exists a potential to improve the moving distance of our algorithms by comparing it with EDA-II. In the future, we plan to focus on researching energy consumption and networks’ lifetime problems.

## Figures and Tables

**Table 1:** Main notations.

Parameters	Value
sensing radius	$R_{s}$ = 6 m, 8 m, ..., 12 m
angle of view (AoV)	AoV = $30^{\circ },60^{\circ },...,180^{\circ }$
size of monitoring area	Area = 100 m ×100 m
the number of sensors, *N*	*N* = 40, 60, 90, ..., 300
